# Hyperbaric Oxygen Therapy in Retinal Arterial Occlusion: Epidemiology, Clinical Approach, and Visual Outcomes

**DOI:** 10.1155/2019/9765938

**Published:** 2019-12-28

**Authors:** Ana Sofia Lopes, Rita Basto, Susana Henriques, Luísa Colaço, Filomena Costa e Silva, Isabel Prieto, Francisco Guerreiro

**Affiliations:** ^1^Department of Ophthalmology of Professor Doutor Fernando Fonseca E.P.E. Hospital (Amadora-Sintra), Lisbon, Portugal; ^2^Underwater and Hyperbaric Medicine Center, Armed Forces Hospital—Lisbon Pole, Lisbon, Portugal

## Abstract

**Purpose:**

To evaluate the efficacy and safety of hyperbaric oxygen therapy (HBOT) in patients with acute retinal artery occlusion (RAO). Secondarily, to analyse the epidemiology and the clinical approach.

**Methods:**

Retrospective study of 13 patients submitted to HBOT between 2013 and 2018. The analysed parameters consisted of: systemic history, time between symptoms onset and treatment, initial approach, number of HBOT sessions, complications of HBOT and best corrected visual acuity—BCVA (of the total sample, central RAO—CRAO—group, and branch RAO—BRAO group).

**Results:**

Arterial hypertension was the most prevalent systemic risk factor (53.8%). Initial therapies were 100% normobaric oxygen administration, topical and oral hypotensive medication, eye massage and aspirin. CRAO was observed in 69.2% and BRAO in 30.8% of the cases, with clinically significant visual improvement (a decrease in logMAR of 0.3) in 55.5% and 75%, respectively. Time between symptoms onset and treatment had a median of 9 hours. The median number of HBOT sessions was 7, without complications.

**Conclusions:**

HBOT provide BCVA improvement in patients with RAO, when it is performed in an early time after the symptom onset. It seems to be an effective and safe therapeutic option for a pathology that still remains without approved treatment.

## 1. Introduction

*Retinal artery occlusion (RAO)* is a rare ophthalmological emergency, with embolism being the most common aetiology [1–4]. It is manifested by acute painless monocular vision loss and a meaningful visual improvement (three lines in Snellen chart) is expected only in 10% of the cases [1–7]. The time of ischemia is classically the critical prognosis factor for the visual outcome, considering that the retina is the tissue of the human body with the highest oxygen consumption, being therefore very sensitive to variations in oxygen [1, 4, 8, 9]. Animal models of central RAO (CRAO) have demonstrated that an ischemic insult to the retinal tissue greater than 90 minutes (min) will lead to some degree of inner retina damage and it will be irreversible if greater than 240 min [10]. As opposed to animal models, in humans there is no clear timeline until irreversible anoxic retinal damage occurs (some authors suggest elapsed time of around 6–6.5 hours, h) [11]. RAO remains without approved treatment and traditional options are not effective enough, having globally a nonsignificant visual outcome improvement [4, 6].

*Hyperbaric oxygen therapy (HBOT)* delivers 100% oxygen by a pressure greater than 1 atmosphere (atm), increasing plasma oxygen transportation and diffusion, with positive effects in vascular perfusion and neuroprotection, in the presence of an adequate vascular supply. Its pathophysiological mechanisms are based on the retinal double vascular supply, in which the choroidal capillary vessels that supply the outer retina will bypass the occlusion and oxygenate inner ischemic retina until spontaneous reperfusion occurs [1, 2, 4, 8]. Normally, under normobaric conditions the choroidal circulation supplies 60% of the oxygen needed by the retina, which increases to 100% under hyperbaric conditions [4]. HBOT also decrease edema and preserve compromised tissue adjacent to ischaemic area [1, 2, 8]. Karaman et al. demonstrated, in mice models of CRAO, that the apoptotic index of retinal inner cells was lower in the group submitted to HBOT in the reperfusion phase than in control group [12]. Also in an animal experiment in a CRAO mice model, Gaydar et al. showed that HBOT diminished cell loss from 58% to 30%, which was related to increased survival of cells in the retinal inner layer [13]. According to the Tenth European Consensus Conference on Hyperbaric Medicine (2016), the European Committee of Hyperbaric Medicine (ECHM) indicate that CRAO cases have Type 2 recommendation (where HBOT is suggested, as it is supported by acceptable levels of evidence) and level C evidence (when the conditions do not allow for proper randomised controlled trials—RCTs—but there is ample and international expert consensus) [14]. The class of recommendation of HBOT by American College of Cardiology/American Heart Association (ACC/AHA) clinical practice guidelines is IIb (where it is not unreasonable to perform procedure/administer treatment) [15]. Also, Undersea and Hyperbaric Medicine Society (UHMS) refer that patients presenting CRAO within 24 h of symptom onset should be considered for HBOT [4].

*The main purpose of this study* was to evaluate the efficacy and the safety of HBOT in patients with RAO. *The secondary purpose* was to analyse the epidemiology and the clinical approach. The efficacy was evaluated by the: analyses of the best corrected visual acuity (BCVA) improvement, number of HBOT sessions and time between symptoms onset and HBOT. The safety was evaluated by the analyses of frequency and severity of complications.

## 2. Case Presentation

### 2.1. Methods

Retrospective study of 13 patients (13 eyes) with RAO from the Professor Doutor Fernando Fonseca E.P.E. Hospital (HFF, Lisbon, Portugal) were submitted to HBOT at the Underwater and Hyperbaric Medicine Center of the Armed Forces Hospital—Lisbon Pole, between September 2013 and June 2018. Inclusion criteria consisted of: RAO documented by fluorescein angiography; absence of patent cilioretinal artery; time between symptoms onset and treatment no longer than 24 h; absence of previous ophthalmic pathology with relevant impact on BCVA; regular follow-up with documented BCVA; and tolerance to HBOT. All consecutive patients meeting the criteria during the period considered were included in the study and this was performed according to the principles of the Helsinki Declaration (2013).

Data was collected for the study through consultation of patients' clinical records. The parameters studied were: demographic (patients' age and sex) and epidemiological factors (personal systemic history, as dyslipidemia, arterial hypertension, diabetes, smoking habits, cerebrovascular or coronary disease, arrhythmia or other relevant factor for arterial occlusion; and type of retinal occlusion between CRAO and branch RAO—BRAO); time between symptoms onset and treatment; the initial approach in the Emergency Room; number of HBOT sessions; complications of HBOT (frequency and severity); and BCVA (total sample, CRAO group and BRAO group). BCVA was analysed before and after each session of HBOT, quantified initially in the Snellen chart in decimal scale and then converted to the logarithm of the minimal angle of resolution (logMAR) scale to perform the statistical analysis. In case of very low vision (<0.05 in Snellen chart), BCVA was assessed by semiquantitative scale, the capability to count-fingers (CF), to see hand movement (HM) or light perception (LP) at a distance of 30 cm. Decimal value attributed to CF, HM and LP was 0.014, 0.005 and 0.0001, respectively, as described in some previous studies [2, 16]. A clinically significant visual improvement was defined as a decrease in logMAR of 0.3 [1, 2, 5].

The HBOT protocol applied had two phases: patients were initially submitted to two daily sessions of 100% oxygen at 2.5 atm during 90 min during the first three consecutive days; then, patients were submitted to additional sessions (1 per day) if BCVA improvement occurred and until it stabilized, or the treatment was interrupted if no subjective BCVA gain occurred after the first three consecutive sessions. This method followed the treatment algorithm of UHMS [4].

A statistical analysis was performed with the SPSS program (Statistical Package for Social Sciences, version 22.0). Continuous variables were presented as median with minimum and maximum values. Wilcoxon signed-rank test was applied to analyse the BCVA variation before and after HBOT. A *p*-value <0.05 was considered to be statistically significant.

### 2.2. Results

**Figure 1:**
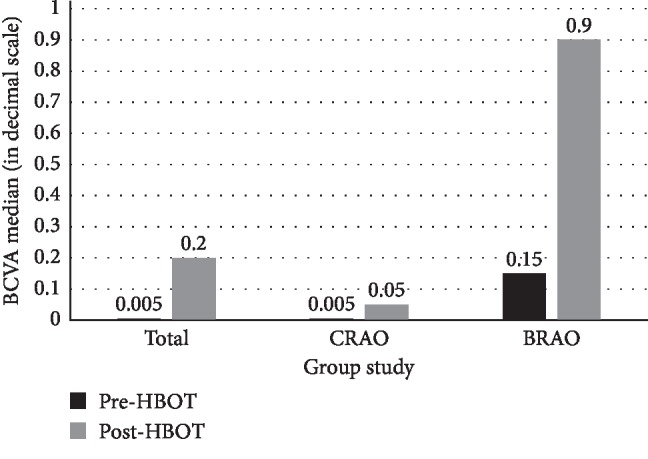
BCVA pre and post-HBOT (in decimal scale).

**Figure 2:**
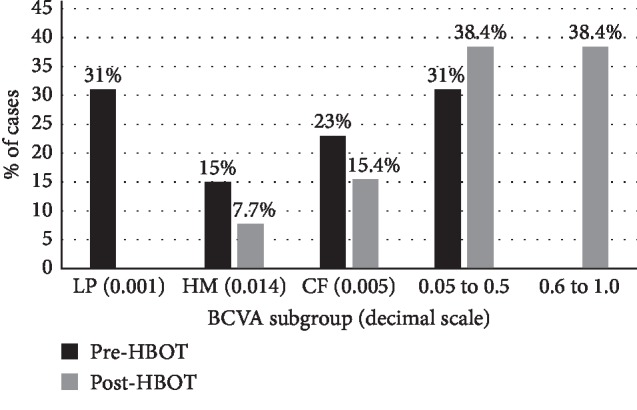
Prevalence of BCVA subgroups (in decimal scale) for the total sample.

(a) Demographic factors: the study included 13 eyes from 13 patients, 8 males and 5 females, with a median age of 70 years (min. 41; max. 83).

(b) Epidemiological factors:

- Personal systemic history: arterial hypertension was the most prevalent (53.8%, number of patients—*n* = 7), followed by cerebrovascular disease (30.8%, *n* = 4), dyslipidemia (23.1%, *n* = 3), coronary disease (7.7%, *n* = 1), arrhythmia (7.7%, *n* = 1) and smoking habits (7.7%, *n* = 1).
- Type of retinal occlusion: CRAO was observed in 69.2% (*n* = 9) of the cases and BRAO in 30.8% (*n* = 4).

(c) Time between symptoms onset and treatment: median of 9 h (min. 2; max. 20), with 77% of cases up to 12 h.

(d) The initial approach in the Emergency Room consisted of: administration of normobaric oxygen in 69.2% (*n* = 9), topical hypotensive application and oral acetazolamide in 53.8% each (*n* = 7), and eye massage and oral administration of aspirin in 46.1% each (*n* = 6).

(e) Number of HBOT sessions: median of 7 (min. 3; max. 18), which corresponds to a total of 10.5 h of treatment.

(f) Complications of HBOT: no complications were observed.

(g) BCVA

- Difference between pre and post-HBOT BCVA: there was a statistically significant improvement in BCVA of the total sample, the CRAO group and the BRAO group, with this one having a better outcome, as detailed in Table 1 and Figure 1.
- Clinically significant improvement of BCVA: it was observed in 61.5% (*n* = 8) of cases in the total sample (*n* = 13). In the group of patients who improved (*n* = 8), 62.5% (*n* = 5) were patients with CRAO and 37.5% (*n* = 3) were patients with BRAO. These results correspond also to an improvement in 55.5% (*n* = 5) of cases in the CRAO group (*n* = 9) and in 75% (*n* = 3) of cases in the BRAO group (*n* = 4). Best results were observed when HBOT was performed within 9 h of symptom onset. The detailed prevalence of BCVA subgroups (in decimal scale) for the total sample is shown in Figure 2.

## 3. Discussion

According to our study, HBOT seems to be an effective and safe option in the treatment of patients with RAO, in case of an early administration. It may be an option to consider also because there is no approved treatment yet for RAO, the current alternative therapies do not have similar outcomes and without treatment there is little chance of visual improvement by the natural history [1, 2, 4].

The results of our study are in agreement with the literature and other studies, as patients responded well to HBOT, showing a clinically significant BCVA improvement in 61.5% (*n* = 8) of cases in the total sample, of which 62.5% (*n* = 5) belongs to the CRAO group and 37.5% (*n* = 3) belongs to the BRAO group [1, 2, 11, 17]. Coelho et al. observed a clinical significant BCVA improvement in 71.4% of the patients following HBOT and Hadanny et al. a BCVA improvement in 67.2% [2, 11]. Hadanny et al. also demonstrated a significant mean improvement in BCVA (logMar) of 0.526 ± 0.688, from 2.14 ± 0.50 to 1.61 ± 0.78 (*p* < 0.01), with 67% of the patients achieving a BCVA gain >0.3 logMar [11]. Also, an improvement from HM to CF has been described to be clinically relevant, and corresponds to an interval of 4 lines (in 0.1 log-unit steps) [2, 16]. Our results are in accordance with these. We observed an evolution of 31% of cases with LP to 0% after treatment, 15% of cases with HM to 7.7% and 23% to 15.4% of cases with CF.

The median number of HBOT sessions is also a parameter that contributes to the analysis of the efficacy of this therapeutic option. The median number of our study, which corresponds to a total of 10.5 h of treatment, is in line with what Wu et al. reported in his recent meta-analysis of seven randomized controlled trials (251 eyes, with the most effective treatment length over 9 hours) [6]. It is also noteworthy that, according to UHMS, the optimum number of HBOT sessions depends on the severity and duration of the patients' symptoms and the degree of response to them. Most patients stabilize within 1 week of symptom onset [4].

Our good results may be related to the early timing between symptoms and treatment (median of 9 h and 77% of cases within 12 h of symptom onset) and, the absence of other ophthalmic pathology. It is well known that the period of time until the treatment is considered the critical factor for the visual prognosis, between the several factors that may influence it. This is based on the main pathophysiological mechanism of an early HBOT, in case of RAO, of the hyperbaric oxygen supply to the inner and outer retinal layers through the choroid [1, 2, 4, 17–19]. However, the best time to perform treatment is controversial. Ideally, the shorter the time delay until treatment, the better the likelihood of recovering ischemic retina that is threatened but viable (penumbra) [4]. Coelho et al., Beiran et al. and Hertzog et al. described that HBOT is most useful up to 8 h of visual loss and Butler et al. documented that the best evidence is a delay up to 12 h of visual loss [2, 5, 9, 20]. The UHMS refer that HBOT should be started in the first 24 h after the onset of the symptoms, and some ophthalmology literature includes strongest evidence for symptomatic improvement in cases with less than 12 hours' delay [4, 9, 21, 22].Despite this traditional critical prognostic factor, Hadanny et al. recently reported that having cherry-red spot (CRS) macula in the fundoscopy at presentation is associated with a prominent VA improvement and a higher success of the treatment, than the patients without CRS at presentation. It was observed a clinically significant visual improvement in 86.0% of patients with CRS compared with 49.4% of patients without CRS at presentation (*p* = 0.0001). In this context, the authors documented that CRS can be used as a marker for irreversible anoxic retinal damage for patients candidates for HBOT. They even highlight that it, rather than the time delay from symptoms onset, should be used as the most important marker for treatment success [11].

The safety of HBOT is another factor that supports this therapeutic option. It is essential to preclude any contraindication to treatment in advance, as well as to perform a regular patient follow-up. In our study, no patient had complications. According to the literature and some similar studies, the most prevalent complication of HBOT is ear barotrauma, which usually have a full spontaneous resolution in few days [2, 4].

We also highlight the type of occlusion as essential factor for visual prognosis, influencing treatment response: BRAO have a better prognosis, as our study showed in the comparison between the CRAO and BRAO groups. Most studies include only cases of CRAO. Wu et al.'s recent meta-analysis included both CRAO and BRAO cases, with good visual outcome in both groups, concluding that oxygen therapy is considered an effective method of treatment for RAO diseases. It reported a global improvement in BCVA between 58.8% and 90%. This study also stands out for including the comparison between a group under HBOT, a group under normobaric oxygen and a group not submitted to oxygen therapy, as well as the result of traditional therapies. Oxygen therapy groups were more likely to have visual improvement (5.61× more); the group under HBOT presented better final visual outcome and with statistical significance; there was no statistical significance between HBOT alone and combined treatment with traditional therapies, nor between the CRAO and BRAO groups (despite both groups presented favorable visual outcomes) [6].

Considering that RAO is the ocular equivalent of an acute cerebral ischemic event by the AHA and American Stroke Association (ASA), having the same risk factors and aetiology, management of RAO should also include secondary prevention of further vascular events, such as cerebral ischemia, myocardial infarction, and cardiovascular death. In this context, RAO may be a “warning sign” for the concomitant evolution of systemic vascular pathologies, thus implying a timely medical articulation with Internal Medicine, Cardiology and/or Neurology. The prevalence of vascular risk factors in our study is consistent with other studies, highlighting the arterial hypertension, dyslipidemia, and brain and cardiovascular diseases [3, 23, 24].

There are still few studies regarding this issue. Many of them have relatively small study samples and some of them differ not only in the definition of the ideal “time window” to perform HBOT but also on the HBOT protocol, regarding that there is no consensus for treatment or guideline-based therapy [1, 2]. Thus, the discussion of this issue may contribute to an improvement of the clinical practice.

*The main limitations of our study* were the retrospective profile and sample size (however, this is a rare pathology and not all patients are indicated for HBOT). *Aspects to highlight* are: the inclusion of CRAO and BRAO cases, considering that most studies include only CRAO cases; and the great impact on clinical practice of this issue, whereas HBOT appears to be a promising therapeutic option in a pathology without an approved effective treatment and with a bad visual prognosis according to its natural history.

*To conclude*, HBOT may improve the BCVA in patients with RAO when it is provided in the first 24 h after the onset of symptoms. Further studies with more patients are necessary to better analyze the value and safety of HBOT in the treatment of RAO, as well as the exact “time window” to perform it.

## Figures and Tables

**Table 1:** BCVA pre and post-HBOT (decimal and logaritmic scales; values in median with range—minimum and maximum values).

		Total	CRAO	BRAO
BCVA pre-HBOT	Decimal scale	0.005 (min. 0.0001; max. 0.2)	0.005 (min. 0.0001; max. 0.014)	0.15 (min. 0.1; max. 0.2)
	Logaritmic scale	2.3 (min. 4; max. 0.7)	2.3 (min. 4; max. 1.85)	0.82 (min.1; max. 0.7)
BCVA post-HBOT	Decimal scale	0.2 (min. 0.005; max. 1)	0.05 (min. 0.005; max. 1)	0.9 (min. 0.6; max. 1)
	Logaritmic scale	0.7 (min. 2.3; max. 0)	1.3 (min. 2.3; max. 0)	0.045 (min. 0.22; max. 0)
*p*-value (^∗^<0.05)		0.007^∗^	0.03^∗^	0.005^∗^

## References

[1] 
Soares A., Gomes N. L., Mendonça L., Ferreira C. (2017). The efficacy of hyperbaric oxygen therapy in the treatment of central retinal artery occlusion. *BMJ Case Reports*.

[2] Coelho P., Ferreira A. P., Gonçalves R. (2018). Hyperbaric oxygen therapy following central retinal artery occlusion—a retrospective case series analysis. *Oftalmologia*.

[3] Dattilo M., Newman N. J., Biousse V. (2018). Acute retinal arterial ischemia. *Annals of Eye Science*.

[4] Murphy-Lavoie H., Butler F., Hagan C. (2012). Central retinal artery occlusion treated with oxygen: a literature review and treatment algorithm. *Undersea & Hyperbaric Medicine*.

[5] Beiran I., Goldenberg I., Adir Y., Tamir A., Shupak A., Miller B. (2001). Early hyperbaric oxygen therapy for retinal artery occlusion. *European Journal of Ophthalmology*.

[6] Wu X., Chen S., Li S. (2018). Oxygen therapy in patients with retinal artery occlusion: a meta-analysis. *PLoS One*.

[7] Hayreh S. S., Zimmerman M. B. (2005). Central retinal artery occlusion: visual outcome. *American Journal of Ophthalmology*.

[8] Pereira C. V., Pina S., Azevedo A. R. (2014). Oclusão arteriolar retiniana e a oxigenoterapia hiperbárica. *Oftalmologia*.

[9] Hertzog L. M., Meyer G. W., Carson S., Strauss M. B., Hart G. B. (1992). Central retinal artery occlusion treated with hyperbaric oxygen. *Journal of Hyperbaric Medicine*.

[10] Hayreh S. S., Zimmerman M. B., Kimura A., Sanon A. (2004). Central retinal artery occlusion, retinal survival time. *Experimental Eye Research*.

[11] Hadanny A., Maliar A., Fishlev G. (2017). Reversibility of retinal ischemia due to central retinal artery occlusion by hyperbaric oxygen. *Clinical Ophthalmology*.

[12] Karaman S., Ozkan B., Yazir Y. (2016). Comparison of hyperbaric oxygen versus iloprost treatment in an experimental rat central retinal artery occlusion model. *Graefe’s Archive for Clinical and Experimental Ophthalmology*.

[13] Gaydar V., Ezrachi D., Dratviman-Storobinsky O., Hofstetter S., Avraham-Lubin B. C., Goldenberg-Cohen N. (2011). Reduction of apoptosis in ischemic retinas of two mouse models using hyperbaric oxygen treatment. *Investigative Ophthalmology & Visual Science*.

[14] Mathieu D., Marroni A., Kot J. (2017). Tenth European consensus conference on hyperbaric medicine: recommendations for accepted and nonaccepted clinical indications and practice of hyperbaric oxygen treatment. *Diving and Hyperbaric Medicine*.

[15] Gibbons R. J., Smith S., Antman E. (2003). American college of Ccardiology; american heart association: american college of cardiology/american heart association clinical practice guidelines: Part I. Where do they come from?. *Circulation*.

[16] Schulze-Bonsel K., Feltgen N., Burau H., Hansen L., Bach M. (2006). Visual acuities “hand motion” and “counting fingers” can be quantified with the Freiburg visual acuity test. *Investigative Opthalmology & Visual Science*.

[17] Cope A., Eggert J., O’Brien E. (2011). Retinal artery occlusion: visual outcome after treatment with hyperbaric oxygen. *Diving and Hyperbaric Medicine*.

[18] Varma D. D., Cugati S., Lee A. W., Chen C. S. (2013). A review of central retinal artery occlusion: clinical presentation and management. *Eye*.

[19] Weiss J. N. (2009). Hyperbaric oxygen treatment of nonacute central retinal artery occlusion. *Undersea & Hyperbaric Medicine*.

[20] Butler F. K., Hagan C., Murphy-Lavoie H. (2008). Hyperbaric oxygen therapy and the eye. *Undersea & Hyperbaric Medicine*.

[21] Li H. K., Dejean B. J., Tang R. A. (1996). Reversal of visual loss with hyperbaric oxygen treatment in a patient with Susac syndrome. *Ophthalmology*.

[22] Yotsukura J., Adachi-Usami E. (1993). Correlation of electroretinographic changes with visual prognosis in central retinal artery occlusion. *Ophthalmologica*.

[23] Dattilo M., Biousse V., Newman N. J. (2017). Update on the management of central retinal artery occlusion. *Neurologic Clinics*.

[24] Sacco R. L., Kasner S. E., Broderick J. P. (2013). An updated definition of stroke for the 21^st^ century: a statement for healthcare professionals from the American Heart Association/American Stroke Association. *Stroke*.

